# Animal Trauma Triage (ATT) Score and Clinical Determinants of Survival in Dogs and Cats with Traumatic Injuries in Thailand

**DOI:** 10.3390/vetsci13050474

**Published:** 2026-05-14

**Authors:** Kritjit Phannithi, Suwicha Kasemsuwan, Narudee Kashemsant, Monchanok Vijarnsorn

**Affiliations:** 1 Emergency and Surgery Units, Kasetsart University Veterinary Teaching Hospital, Faculty of Veterinary Medicine, Kasetsart University, Bangkok 10900, Thailand; kritjit.p@ku.th; 2 Department of Veterinary Public Health, Faculty of Veterinary Medicine, Kasetsart University, Bangkok 10900, Thailand; fvetswk@ku.ac.th; 3 Department of Physiology, Faculty of Veterinary Medicine, Kasetsart University, Bangkok 10900, Thailand; narudee.k@ku.th; 4 Department of Companion Animal Clinical Sciences, Faculty of Veterinary Medicine, Kasetsart University, Bangkok 10900, Thailand

**Keywords:** animal trauma, mortality, prognostic factors, veterinary emergency medicine, acid–base status, ionized calcium

## Abstract

Trauma is a major cause of emergency presentation in small animal practice, and accurate early assessment is essential for prognosis. The Animal Trauma Triage (ATT) score is widely applied in Western veterinary settings but has been less frequently evaluated in Asian veterinary institutions. This prospective observational study assessed the prognostic value of the ATT score and of relevant clinical variables in 184 dogs and cats presenting with traumatic injuries to a university veterinary teaching hospital in Thailand. ATT scores, clinicopathological parameters, and management variables were analyzed in relation to survival outcome. The overall mortality rate was 35.3%. Higher ATT scores, lower blood pH, lower ionized calcium concentrations, and increasing age were independently associated with non-survival (*p* < 0.05). An ATT score ≥ 5 was associated with increased odds of non-survival (OR = 4.207, 95% CI: 1.903–9.301), yielding a sensitivity of 86.2% and specificity of 40.3% for identifying high-risk patients. Among animals with documented surgical indications, those that did not undergo surgery demonstrated higher mortality than those receiving surgical intervention; however, this finding should be interpreted cautiously because treatment allocation was influenced by clinical stability and owner-related factors. These results demonstrate the clinical usefulness of the ATT score as a triage instrument when interpreted in context with clinical laboratory parameters, age, and treatment responses.

## Simple Summary

Traumatic injury is a major cause of emergency hospitalization and death in dogs and cats. Rapid assessment of injury severity is essential to improve survival. The Animal Trauma Triage (ATT) score is a clinical tool widely used in Western countries to assess trauma severity and estimate the probability of survival. However, its use and validation in veterinary emergency clinics in Thailand remain limited. In this study, we evaluated the ATT score in dogs and cats treated at the reporting institution’s veterinary teaching hospital. ATT scores were correlated with a higher risk of death. In addition to the ATT score, increasing age, acidosis, lower blood calcium levels, and surgery indicated but not performed were also negatively associated with survival. These findings support the predictive value of the ATT score in this veterinary emergency hospital setting and highlight additional factors that may assist veterinarians in early risk assessment and clinical decision-making for trauma patients.

## 1. Introduction

Traumatic injuries are among the leading causes of emergency presentation in small animal practice and frequently require immediate veterinary attention [1]. Trauma also represents a major cause of morbidity and mortality in dogs and cats, as demonstrated in recent veterinary trauma studies and registry-based analyses [2,3,4]. Trauma can be categorized according to the type of injury as blunt, penetrating, or a combination thereof [5,6], and their severity ranges from mild to life-threatening, requiring accurate evaluation and timely intervention to improve survival outcomes. Accurate early assessment and timely intervention are therefore critical to improving survival outcomes. In emergency settings, reliable triage and prognostic tools are essential to support clinical decision-making, optimize resource allocation, and facilitate communication with owners [5,7].

Trauma scoring systems have been developed to standardize severity assessment, characterize patient populations, and enable outcome benchmarking across institutions [7,8,9]. Most scoring systems assign numerical values to injury severity to estimate the likelihood of survival to discharge and to guide evidence-based management decisions [10]. Earlier approaches to trauma assessment included qualitative classification of injury severity into categories such as mild, moderate, severe, and catastrophic, as well as the Modified Glasgow Coma Scale (MGCS) for assessment of head trauma severity. Among currently available tools, the Animal Trauma Triage (ATT) score, originally developed by Rockar and Drobatz [11], was designed for dogs and cats to evaluate trauma severity and estimate survival probability. The ATT score assesses parameters of six physiologic systems—perfusion, cardiovascular, respiratory, muscular/integumentary/ocular, skeletal, and neurologic—each graded from 0 (no injury) to 3 (critical injury), yielding a maximum score of 18. The ATT score has been validated in multicenter studies and is widely used in veterinary trauma centers across North America and Europe, with substantial evidence supporting its role in triage and outcome prediction [6,7,9,10,11,12,13,14,15,16,17,18,19,20,21,22,23,24,25,26].

Despite its international adoption, clinical validation of the ATT score in Southeast Asian veterinary settings remains limited. Differences in case characteristics, referral patterns, resource availability, and clinical practice environments may influence prognostic performance across regions. Accordingly, the objective of this study was to evaluate the prognostic value of the ATT score in dogs and cats with traumatic injuries treated at a single veterinary teaching hospital in Thailand. In addition, we attempted to identify clinical and physiologic variables independently associated with non-survival, thereby providing region-specific data to improve trauma assessment and emergency care practices.

## 2. Materials and Methods

### 2.1. Study Population, Study Design, and Ethical Approval

This prospective observational study included dogs and cats presenting with traumatic injuries to the Emergency Unit of the reporting institution, during the year 2023. All animals underwent standardized trauma assessments and received emergency care upon admission.

The study protocol was approved by the Kasetsart University Institutional Animal Care and Use Committee (approval no. ACKU68-VET-051). Informed consent was obtained from all owners prior to inclusion.

### 2.2. Case Selection

Cases were eligible for inclusion if complete medical records were available, including documentation of the Animal Trauma Triage (ATT) score, species-specific pain assessment using the Colorado State University Canine Acute Pain Scale (CSU-CAP) for dogs or Feline Acute Pain Scale (CSU-FAPS) for cats, body condition score (BCS), and relevant laboratory parameters obtained at hospital admission, and verifiable survival status within seven days following presentation. Only cases meeting all inclusion criteria were entered into the final analysis.

### 2.3. Clinical and Laboratory Data Collection

Prior to study initiation, all emergency clinicians participated in a standardized training session on ATT scoring criteria to ensure consistent interpretation. ATT scores were routinely reviewed by attending emergency veterinarians to promote scoring consistency.

Pain was assessed using the CSU-CAP in dogs [27] and CSU-FAPS in cats [28], both routinely applied by trained clinicians at the institution [29]. Body condition score (BCS) was recorded according to standardized species-specific scales: cats were categorized as underweight (BCS 1–4), ideal (BCS 5), or overweight (BCS 6–9); dogs were categorized as underweight (BCS 1–3), ideal (BCS 4–5), or overweight (BCS 6–9) [30,31,32]. Treatment type was recorded as surgical or non-surgical. Surgical treatment was defined as a major operative procedure performed under general anesthesia. Additional recorded variables included age, sex, time from injury to presentation, previous treatment at another facility, presence of pre-existing disease, results of physical examination, length of hospitalization, and total treatment cost. Trauma was classified by mechanism (blunt, penetrating, or combined) [6] and injury pattern (single vs. multiple injuries).

### 2.4. Laboratory Methods

Blood samples were collected in ethylenediaminetetraacetic acid (EDTA) tubes and serum separator tubes as early as possible upon hospital arrival, using either venipuncture or intravenous catheter placement, depending on patient condition. All samples were submitted to the in-house KUVTH laboratory within 15 min of collection. Hematologic analyses (hematocrit, plasma protein, white blood cell count, platelet count) were performed using an automated hematology analyzer (XN-1000 Vet, Sysmex, Kobe, Japan). Serum biochemistry (blood urea nitrogen [BUN], creatinine, alanine aminotransferase [ALT], total protein, albumin) was measured using an automated chemistry analyzer (iLab Taurus, Instrumentation Laboratory, Werfen Group, Barcelona, Spain). Blood samples for blood gas analysis (pH, bicarbonate, sodium, potassium, chloride, ionized calcium, glucose, lactate) were collected using commercially available heparinized blood gas syringes and analyzed immediately in the emergency unit using a point-of-care analyzer (GEM Premier 3500, Instrumentation Laboratory, Werfen Group, Barcelona, Spain).

### 2.5. Statistical Analyses

Data were entered into Microsoft Excel (Microsoft, Redmond, WA, USA) and analyzed using NCSS software (version 20.0.6; NCSS, LLC, Kaysville, UT, USA) and R software (version 4.5.3; R Foundation for Statistical Computing, Vienna, Austria).

Normality was assessed using the Kolmogorov–Smirnov test. Normally distributed continuous variables are presented as mean ± standard deviation (SD), whereas non-normally distributed variables are reported as median with interquartile range (IQR). Categorical variables are presented as frequencies and percentages with 95% CI.

Group comparisons (survival vs. non-survival) were performed using Student’s *t*-test or Welch’s *t*-test for normally distributed data and the Mann–Whitney U test for non-normally distributed data. Categorical variables were analyzed using Pearson’s chi-square test or Fisher’s exact test, as appropriate. Spearman’s rank correlation coefficient was used to evaluate associations between ATT score and hospitalization duration or treatment cost.

Variables with *p* < 0.20 in univariable analysis were assessed for collinearity prior to multivariable modeling. Collinearity was defined as Spearman’s correlation coefficient ≥ 0.60 (*p* < 0.05). Variance inflation factors (VIFs) were calculated, with VIF < 5 considered acceptable. Prior to logistic regression analysis, blood pH and ionized calcium were rescaled to 0.1-unit increments so that odds ratios reflected changes per 0.1-unit increase. A manual stepwise selection approach was applied to construct the multivariable logistic regression model identifying independent predictors of non-survival. Statistical significance was set at *p* < 0.05. Model calibration of the final multivariable model was assessed using the Hosmer–Lemeshow goodness-of-fit test, with animals grouped into deciles based on predicted probabilities.

Receiver operating characteristic (ROC) curve analysis was performed to evaluate the discriminative performance of the ATT score. The area under the curve (AUC) and optimal cutoff threshold were determined. The association between the selected ATT threshold and non-survival was further evaluated using logistic regression to calculate adjusted odds ratios (ORs) with 95% confidence intervals.

In addition, an exploratory post hoc analysis was conducted to evaluate survival outcomes according to surgical management status and to compare clinical and laboratory parameters between animals with surgical indications who underwent surgery and those managed non-surgically. As these comparisons were exploratory and hypothesis-generating in nature, no adjustment for multiple comparisons was applied.

A secondary analysis was performed to compare baseline characteristics between included and excluded cases using variables available at presentation. Continuous variables were compared using the Mann–Whitney U test, and categorical variables were compared using the chi-square or Fisher’s exact test, as appropriate.

To address potential treatment-allocation bias, two sensitivity analyses were conducted. First, the multivariable model was repeated without the treatment variable. Second, animals with documented surgical indications that were managed medically were excluded, and the multivariable model was re-run in the reduced cohort.

Internal validation was performed using bootstrap resampling (1000 iterations) of the multivariable logistic regression model, including all candidate variables retained after univariable screening and collinearity assessment.

## 3. Results

### 3.1. Study Population and Clinical Characteristics

**Figure 1:**
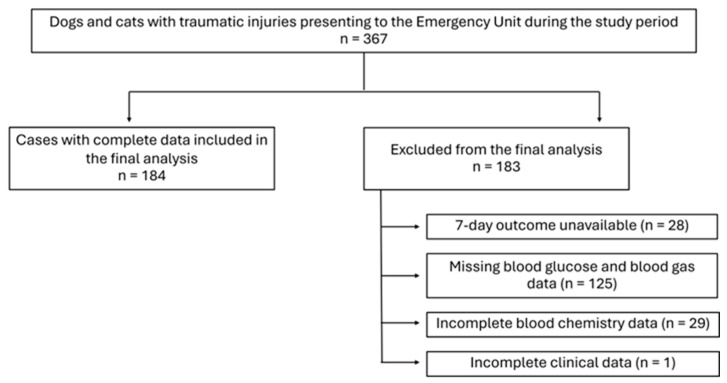
Flow diagram of case inclusion and exclusion in the study population.

During the study period, 367 dogs and cats with traumatic injuries were admitted to the emergency unit. After exclusion of 183 cases due to incomplete or missing data, 184 cases were included in the analysis. A study flow diagram summarizing case inclusion and exclusion is shown in Figure 1.

Comparison of available baseline characteristics between included and excluded cases is provided in Supplementary Table S1. The two groups were similar with respect to several variables, including age and treatment category. However, excluded cases had lower ATT scores and pain scores, a lower frequency of multiple-trauma presentations, and shorter duration of hospitalization and lower treatment costs.

Of these, 83 (45.1%) were dogs and 101 (54.9%) were cats. Among dogs, 47 (56.6%) were male and 36 (43.4%) were female; among cats, 55 (54.5%) were male and 46 (45.5%) were female.

**Figure 2:**
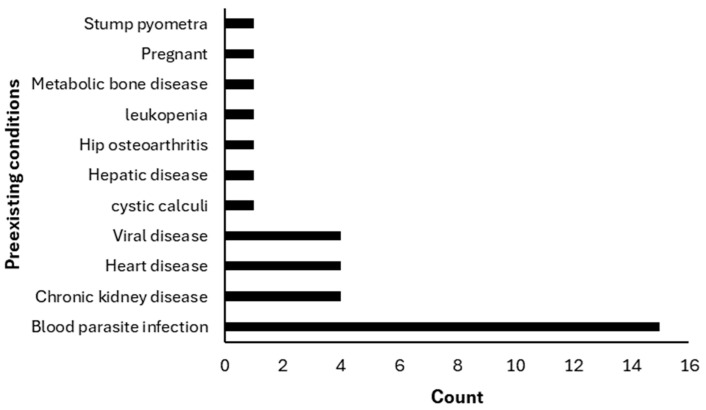
Distribution of pre-existing conditions among trauma patients (*n* = 34) with documented comorbidities. Percentages are calculated relative to the subgroup of animals with pre-existing disease (18.5% of the total study population, *n* = 184).

The median age was 36 months, and the median time from injury to presentation at KUVTH was 285 min. Prior treatment at another facility had been administered in 80 cases (43.5%), while 104 (56.5%) had not received prior care. Pre-existing conditions were identified in 34 animals (18.5%) (Figure 2).

Body condition score (BCS) distribution was 26.6% (49/184) underweight, 43.5% (80/184) ideal, and 29.9% (55/184) overweight. Pain assessment using CSU-CAP and CSU-FAPS yielded scores of 1 in 16.9%, 2 in 42.4%, 3 in 21.2%, and 4 in 19.6% of cases.

### 3.2. Trauma Profile and Injury Mechanism

Blunt trauma accounted for 76.6% (141/184) of cases, whereas 21.2% (39/184) sustained penetrating injuries. The remaining cases involved combined or unspecified mechanisms. The most common causes of trauma were vehicular accidents (67.9%), dog bites (22.8%), and falls from height (7.1%).

Multiple injuries were present in 121 animals (65.8%), while 63 (34.2%) sustained a single injury.

### 3.3. Therapeutic Approaches and Outcomes

Of the 184 cases, 101 (54.9%) were managed non-surgically and 83 (45.1%) underwent surgical intervention. Surgical procedures are summarized in Table 1 and categorized by affected system.

Overall, 119 animals (64.7%) survived to discharge, whereas 65 (35.3%) did not.

Among the non-surgical group, 40 of 101 animals (39.6%) had documented clinical indications for surgical intervention but did not undergo surgery. In this subgroup, 34 (85.0%) did not survive and 6 (15.0%) survived. In contrast, among non-surgical cases without surgical indication, 15 (24.6%) did not survive and 46 (75.4%) survived (*p* < 0.001).

### 3.4. Univariate Statistical Analysis

Univariable analysis identified prior treatment, pain score, trauma type, and treatment category (surgical vs. non-surgical) as associated with survival outcome (*p* < 0.20). Sex, species, pre-existing disease, BCS, and injury multiplicity were not associated with outcome at this threshold (Table 2).

### 3.5. Multivariable Logistic Regression Analysis

Variables meeting the univariable screening threshold (*p* < 0.20) were evaluated for multicollinearity prior to inclusion in the multivariable model. Moderate to strong correlations were observed among blood pH, bicarbonate, base excess of the extracellular fluid (BEecf) and lactate (*r* = 0.52–0.96, *p* < 0.001), as well as between blood urea nitrogen (BUN) and creatinine (*r* = 0.79, *p* < 0.001). To minimize redundancy, only blood pH and BUN were retained for multivariable modeling based on stronger univariable associations (*p* < 0.001).

The initial multivariable logistic regression model incorporated age, ATT score, blood pH, potassium, ionized calcium, hematocrit, BUN, albumin, prior treatment, pain score category, trauma type, and treatment category (non-surgical vs. surgical). Following manual stepwise selection, five variables remained independently associated with non-survival (Table 3). Although pain score was associated with outcome in univariable analysis, it was not retained in the final multivariable model after stepwise selection because it did not provide independent prognostic information after adjustment for ATT score and other physiologic variables.

Lower blood pH was associated with increased odds of non-survival (OR = 0.544, 95% CI: 0.358–0.828, *p* = 0.003), as were lower ionized calcium concentrations (OR = 0.547, 95% CI: 0.358–0.836, *p* = 0.003). Increasing ATT score (OR = 1.445, 95% CI: 1.156–1.806, *p* = 0.001) and increasing age (per month) (OR = 1.016, 95% CI: 1.007–1.025, *p* < 0.001) were also independently associated with non-survival. In addition, non-surgical management was associated with higher odds of non-survival compared with surgical intervention (OR = 2.963, 95% CI: 1.836–4.780, *p* < 0.001).

The final multivariable model demonstrated good discriminative performance, with an area under the curve (AUC) of 0.870 (95% CI: 0.790–0.922). All retained variables demonstrated acceptable variance inflation factors (VIF < 5), and no significant interaction terms were identified. Model calibration was assessed using the Hosmer–Lemeshow goodness-of-fit test. The model demonstrated acceptable calibration, with no evidence of a significant lack of fit (χ^2^ = 12.44, *df* = 8, *p* = 0.133), indicating good agreement between predicted and observed outcomes across all probability strata.

To assess the potential influence of treatment-allocation bias, two sensitivity analyses were performed. First, when the treatment variable was removed from the multivariable model, the principal predictors of non-survival, including age, ATT score, blood pH, and ionized calcium concentration, remained materially unchanged. Second, after excluding animals with documented surgical indications that were managed medically (*n* = 40), the multivariable model included 144 animals. In this analysis, increasing age (OR = 1.019, 95% CI: 1.008–1.031, *p* < 0.001) and lower ionized calcium concentration (OR = 0.525, 95% CI: 0.283–0.971, *p* = 0.029) remained independently associated with non-survival, whereas the associations of ATT score and blood pH were attenuated and no longer statistically significant. The association between non-surgical management and non-survival was attenuated compared with the original model (OR = 1.74, 95% CI: 0.988–3.053, *p* = 0.050) and was no longer statistically significant.

Internal validation using bootstrap resampling (1000 iterations) demonstrated generally stable coefficient estimates, with the principal predictors (age, ATT score, blood pH, ionized calcium, and non-surgical management) showing consistent direction and comparable magnitudes across resampled datasets (Supplementary Table S2).

### 3.6. Predictive Value of the ATT Score

**Figure 3:**
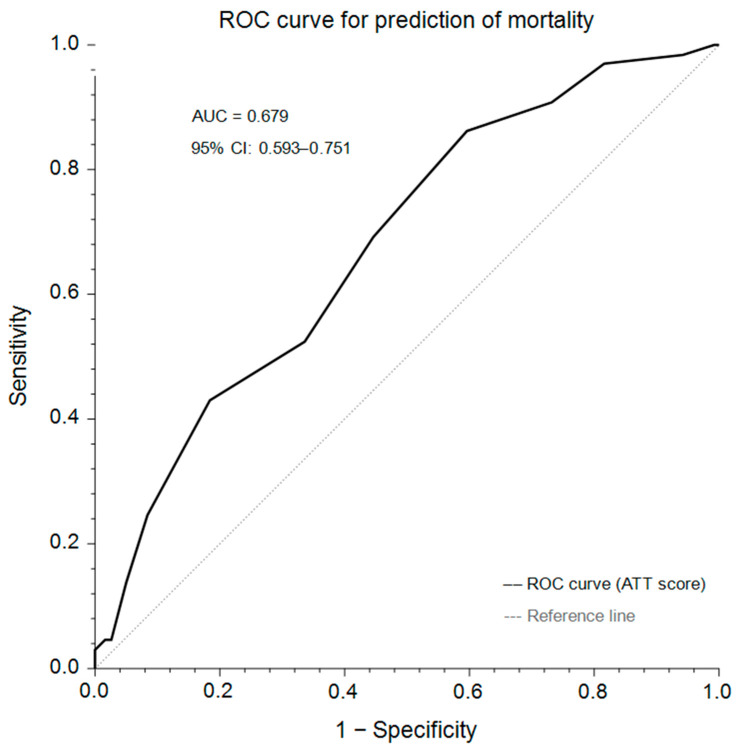
Receiver operating characteristic (ROC) curve of the Animal Trauma Triage (ATT) score for predicting non-survival in dogs and cats with traumatic injuries.

Receiver operating characteristic (ROC) analysis demonstrated that the ATT score was significantly associated with mortality (AUC = 0.679, 95% CI: 0.593–0.751, *p* < 0.001) (Figure 3).

An ATT threshold ≥5 yielded a sensitivity of 86.2% and specificity of 40.3%. Increasing the threshold to ≥7 reduced sensitivity (52.3%) while increasing specificity (66.4%). A threshold ≥10 demonstrated high specificity (95.0%) but low sensitivity (13.9%).

Mortality was higher in animals with ATT ≥ 5 compared to those with ATT < 5 (44.1% vs. 15.8%, *p* < 0.001). Logistic regression confirmed ATT ≥ 5 as a significant predictor of non-survival (OR = 4.207, 95% CI: 1.903–9.301, *p* < 0.001).

### 3.7. Exploratory Post Hoc Analysis of Surgical Management

An exploratory post hoc analysis was performed to further examine the relationship between surgical management and survival outcome. Among the 184 included animals, 83 (45.1%) underwent surgical intervention, whereas 101 (54.9%) were managed without surgery. Of the non-surgically managed cases, 40 animals (39.6%) had documented clinical indications for surgical intervention but did not undergo surgery.

**Figure 4:**
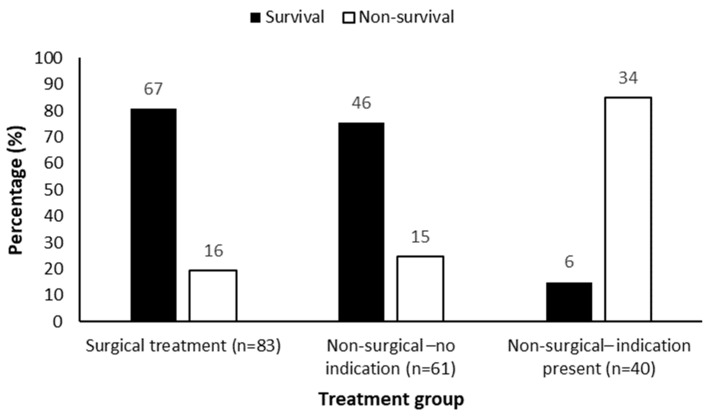
Survival distribution by treatment category (exploratory post hoc analysis) among animals with documented surgical indications. Numbers within bar segments indicate the number of animals in each outcome category.

Animals that did not receive indicated surgical treatment demonstrated a markedly higher non-survival rate compared with those that underwent surgery (85.0% vs. 19.3%, respectively; *p* < 0.001). Survival outcomes according to surgical intervention status are illustrated in Figure 4.

When animals that underwent surgery were compared with those that had clinical indications for surgery but did not receive surgical treatment, several physiological differences were observed. The non-surgical group exhibited higher lactate and blood urea nitrogen concentrations, and lower blood pH, bicarbonate, and albumin levels. Median ATT scores did not differ significantly between groups, suggesting comparable overall injury severity (Table 4). In addition, animals in the surgical group had significantly longer hospitalization durations and higher treatment costs compared with the non-surgical group (both *p* < 0.001).

Within the surgical cohort, procedures were categorized into seven surgical systems (multisystem, thoracic, abdominal, orthopedic, urogenital, integumentary, and maxillofacial). Multisystem procedures were most common and were associated with longer anesthesia duration, operative time, and higher treatment costs. Surgical timing (≤24 h vs. >24 h) differed significantly across surgical systems (*p* < 0.001), and overall timing was associated with survival outcome (*p* = 0.002). However, no significant association between surgical system and survival was observed when timing was analyzed within each surgical category.

These findings should be interpreted cautiously, as treatment allocation was influenced by clinical stability and owner-related factors. This exploratory analysis was performed to provide additional clinical context regarding outcome variability beyond injury severity scoring. No adjustment for multiple comparisons was applied; therefore, the post hoc comparisons should be considered hypothesis-generating rather than confirmatory.

## 4. Discussion

This study evaluated the prognostic performance of the Animal Trauma Triage (ATT) score in dogs and cats presenting with traumatic injuries to a veterinary teaching hospital in Thailand. The ATT score demonstrated moderate discriminative ability for predicting non-survival and remained independently associated with mortality after adjustment for relevant clinical and physiological variables. To our knowledge, this represents one of the first evaluations of ATT performance in a Southeast Asian veterinary emergency setting, providing region-specific data on its prognostic utility.

Consistent with previous multicenter studies conducted in North America and Europe, higher ATT scores were associated with increased mortality risk [2,7,8,9,10,11,33,34]. In our cohort, each one-point increase in ATT score was associated with a 1.45-fold increase in the odds of non-survival. Although the magnitude of effect was slightly lower than that reported in some earlier studies [7,11,21,22,25,26], the direction and clinical relevance of the association were consistent. Differences in case mix, referral patterns, time to presentation, and institutional management protocols may contribute to variation in effect estimates across geographic regions.

Threshold analysis demonstrated that an ATT score ≥ 5 was associated with increased odds of non-survival and yielded high sensitivity (86.2%) but limited specificity (40.3%). This performance profile suggests that the threshold is more suitable for early identification of high-risk patients than for definitive prognostication. In emergency settings, where delayed recognition of critical cases may have substantial consequences, prioritizing sensitivity may be appropriate. However, the ATT score should be interpreted alongside comprehensive clinical assessment rather than used as a standalone determinant of management decisions.

In addition, the discriminative performance of the full multivariable model was substantially higher than that of the ATT score alone (AUC = 0.870, 95% CI: 0.790–0.922 vs. AUC = 0.679, 95% CI: 0.593–0.751), indicating that prognostic performance improved when ATT was interpreted in conjunction with additional physiological and clinical variables. Although the discriminative performance of ATT alone was moderate, this is consistent with prior trauma studies [2,7,8,9,10,11,33,35] and reflects the inherently multifactorial nature of injury outcomes, in which no single scoring system fully captures prognostic complexity. Interestingly, pain score was associated with outcome in univariable analysis but did not remain an independent predictor after multivariable adjustment, suggesting that its prognostic signal may overlap with broader indicators of injury severity, particularly the ATT score, as both variables capture different aspects of trauma burden and physiological stress.

In our cohort, the median age was 36 months, which is consistent with prior trauma studies reporting that small-animal trauma patients are often young [10,16,26,36,37,38,39]. Despite this, increasing age was associated with a greater likelihood of non-survival in our multivariable analysis. This relationship is biologically plausible, as older animals typically have reduced physiological reserve and may carry subclinical comorbidities that limit their ability to compensate following acute injury [37].

Lower blood pH at presentation was independently associated with mortality, underscoring the prognostic importance of acid–base status in trauma patients. Because pH was analyzed per 0.1-unit decrement, the estimated effect size reflects clinically meaningful changes within the narrow physiological range. Acidemia reflects inadequate tissue perfusion and oxygen delivery, leading to anaerobic metabolism and lactate accumulation. Progressive metabolic acidosis may further impair myocardial function, vascular tone, and cellular metabolism, thereby contributing to clinical deterioration. Similar associations between acid–base imbalance and mortality have been reported in both veterinary and human trauma populations [25,37]. These findings emphasize the clinical importance of early detection of hypoperfusion and prompt intervention. Timely restoration of systemic circulation, oxygen delivery, and adequate ventilation may help mitigate the progression of metabolic and respiratory acidosis and improve the probability of survival.

Ionized calcium was also independently associated with outcome. Because ionized calcium was analyzed per 0.1 mmol/L increment, the estimated effect size reflects clinically meaningful changes within the physiological range. As the biologically active fraction of circulating calcium, ionized calcium plays essential roles in myocardial contractility, vascular tone regulation, and coagulation [33,40,41]. Hypocalcemia has been linked to increased mortality and greater need for intensive supportive interventions in both veterinary and human critical care settings [22,33,40,41]. These findings collectively suggest that early physiological derangement, in addition to structural injury severity, substantially influences trauma prognosis.

Surgical management was associated with increased survival in this cohort, a finding consistent with previous reports [7,22,42,43]. However, this association should be interpreted with caution, as treatment allocation in this study was not randomized and was influenced by clinical instability, financial limitations, and owner-related decisions. Therefore, the treatment variable likely reflects not only treatment modality but also clinical feasibility and case severity. This interpretation is supported by the sensitivity analyses. When the treatment variable was removed from the model, the principal prognostic factors remained unchanged, supporting the robustness of the main associations. However, after excluding animals with documented surgical indications that were managed medically, the magnitude of the association between non-surgical management and non-survival was attenuated (from OR 2.96 to 1.74) and was no longer statistically significant. This finding suggests that part of the observed association in the original model may have been influenced by treatment-allocation bias. These findings reinforce that treatment-related context can meaningfully affect model behavior and should be considered when interpreting prognostic estimates.

The overall mortality rate in this study (35.3%) was higher than rates reported in several previous studies (8–25%) [10,11,36,44]. Differences in injury patterns, case severity, referral timing, and healthcare system dynamics may contribute to this variation. Notably, no cases of euthanasia were performed in this cohort. Consequently, all non-surviving cases in the present study likely represent trauma-related mortality rather than elective termination; however, this distinction should be considered when comparing mortality estimates across studies.

Several limitations should be acknowledged. This was a single-center study conducted at a large veterinary teaching hospital, and findings may not be directly applicable to other practice settings. Survival to discharge was used as the primary outcome, which is commonly applied in veterinary trauma studies and reflects a clinically relevant endpoint during the acute phase [6,7,9,17]. However, this definition may not fully capture post-discharge mortality. Animals discharged with guarded prognosis could have died shortly after leaving the hospital and would have been classified as survivors. In addition, loss to follow-up after initial stabilization may further limit outcome accuracy. The 7-day follow-up window was selected to capture early post-traumatic and perioperative mortality [6], although longer-term outcomes were not assessed.

Approximately half of initially admitted trauma cases were excluded because of incomplete data, which may have introduced selection bias. Comparison between included and excluded cases suggested that excluded animals generally had lower ATT and pain scores, fewer multiple-trauma presentations, shorter hospitalization durations, and lower treatment costs, indicating that less severely affected animals may have been less likely to undergo comprehensive diagnostic testing and therefore more likely to have incomplete datasets. As a result, the final analytic cohort may overrepresent more severely affected or more extensively evaluated cases, which may limit the representativeness of the study population. Although multivariable modeling identified independent predictors of outcome, the sample size and number of events may still limit model stability. Model calibration assessed by the Hosmer–Lemeshow goodness-of-fit test demonstrated acceptable agreement between predicted and observed outcomes, and bootstrap resampling showed generally stable coefficient estimates, providing some reassurance regarding model performance within the present dataset. However, these findings do not replace the need for external validation. Additionally, the exploratory post hoc analysis of surgical management should be interpreted cautiously and requires confirmation in prospective studies. Further multicenter investigations are needed to externally validate these findings and refine trauma prognostication strategies across diverse veterinary healthcare settings.

Overall, the findings support the use of the ATT score as a practical triage instrument in veterinary emergency settings when interpreted in conjunction with physiological assessment and clinical context. More broadly, this study reinforces that trauma prognostication is inherently multifactorial, integrating measurable injury burden, dynamic physiological response, and contextual treatment factors within diverse healthcare environments. Continued evaluation across institutions and geographic regions will be essential to refine trauma risk stratification and ensure contextual applicability of prognostic tools.

## 5. Conclusions

In this study, the Animal Trauma Triage (ATT) score demonstrated moderate discriminative ability for predicting non-survival in dogs and cats with traumatic injuries treated at a veterinary teaching hospital in Thailand. An ATT threshold of ≥5 was associated with increased mortality risk and may serve as a practical tool for early identification of high-risk patients when interpreted alongside comprehensive clinical assessment.

Beyond injury severity scoring, age, blood pH, ionized calcium concentration, and treatment allocation were independently associated with outcome. These findings highlight that trauma prognosis reflects not only structural injury burden but also physiological status and management feasibility.

## Figures and Tables

**Table 1:** Distribution of surgical procedures performed among trauma patients undergoing operative treatment (*n* = 83).

Surgery Type	*n*	Percentage (%)
Multisystem surgery	30	36.1
Orthopedic surgery	17	20.5
Urogenital surgery	11	13.3
Thoracic surgery	10	12.1
Abdominal surgery	7	8.4
Integument surgery	5	6.0
Maxillofacial surgery	3	3.6
Total	83	100

**Table 2:** Comparison of clinical and laboratory variables at hospital admission between surviving and non-surviving dogs and cats with traumatic injuries.

Variable	Non-Survival (*n* = 65)	Survival (*n* = 119)	*p*-Value
**Age**	**48 (14–118)**	**36 (12–66)**	**0.061**
Time to treatment	285 (122.5–1008)	217 (114–591)	0.222
**Animal traumatic triage (ATT) score**	**7 (5.00–8.50)**	**5 (3.00–7.00)**	**<0.001**
**Blood pH**	**7.24 (7.17–7.32)**	**7.3 (7.24–7.37)**	**<0.001**
Sodium (mEq/L)	145 (142–148)	145 (142–148)	0.963
**Potassium (mEq/L)**	**4 (3.60–5.25)**	**4 (3.60–4.50)**	**0.102**
**Ionized calcium (mmol/L)**	**1.19 (1.07–1.24)**	**1.25 (1.18–1.30)**	**0.001**
**Lactate (mmol/L)**	**4.2 (2.60–7.60)**	**3 (2.10–4.70)**	**0.007**
**Bicarbonate (HCO_3_^−^) (mmol/L)**	**15.9 (12.90–19.60)**	**18.8 (15.50–20.70)**	**0.004**
BEecf (mmol/L) *	**−11.6 (−15.00 to −6.60)**	**−7.6 (−11.70 to −5.10)**	**0.001**
**Hematocrit (%)**	**32.77 ± 9.19**	**36.23 ± 9.54**	**0.018**
Plasma protein (gm%)	6.60 (6.00–7.40)	7.00 (6.00–7.60)	0.301
White blood cell (×10^3^/µ/L)	16.81 (10.69–24.61)	17.12 (11.74–24.80)	0.613
Platelet (×10^3^/µ/L)	258.46 ± 133.56	266.06 ± 122.37	0.697
**Blood Urea Nitrogen (mg/dL)**	**39.00 (29.00–54.00)**	**28.50 (21.25–38.00)**	**<0.001**
**Creatinine (mg/dL)**	**1.59 (1.20–2.29)**	**1.30 (1.00–1.88)**	**0.006**
Alanine Aminotransferase (U/L)	136 (89.5–325.5)	130 (61.00–382.00)	0.855
**Albumin (g/dL)**	**2.64 ± 0.48**	**2.93 ± 0.43**	**<0.001**
Glucose (mg/dL)	190 (116.00–267.50)	160 (108.00–237.00)	0.328
Species			0.472
Dogs	27/83 (32.5)	56/83 (67.5)	
Cats	38/101 (37.6)	63/101 (62.4)	
Sex			0.749
Female	30/82 (36.6)	52/82 (63.4)	
Male	35/102 (34.3)	67/102 (65.7)	
**Prior treatment**			**0.140**
**No**	**32/104 (30.8)**	**72/104 (69.2)**	
**Yes**	**33/80 (41.3)**	**47/80 (58.8)**	
Body condition scores (BCS}			0.399
Underweight	20/49 (40.8)	29/49 (59.2)	
Ideal weight	24/80 (30.0)	56/80 (70.0)	
Overweight	21/55 (38.2)	34/55 (61.8)	
**Pain score**			**0.013**
**Pain score 1**	**9/31 (29.0)**	**22/31 (71.0)**	
**Pain score 2**	**20/78 (25.6)**	**58/78 (74.4)**	
**Pain score 3**	**16/39 (41.0)**	**23/39 (59.0)**	
**Pain score 4**	**20/36 (55.6)**	**16/36 (44.4)**	
**Type of trauma**			**0.080**
**Blunt trauma**	**45/141 (31.9)**	**96/141 (68.1)**	
**Penetrate trauma and both**	**20/43 (46.5)**	**23/43 (53.5)**	
Condition of trauma			0.809
Single trauma	23/63 (36.5)	40/63 (63.5)	
Multiple trauma	42/121 (34.7)	79/121 (65.3)	
**Treatment**			**<0.001**
**Non-surgical treatment**	**49/101 (48.5)**	**52/101 (51.5)**	
**Surgical treatment**	**16/83 (19.3)**	**67/83 (80.7)**	
Preexisting disease			0.997
No	53/150 (35.3)	97/150 (64.7)	
Yes	12/34 (35.3)	22/34 (64.7)	

Note: Variable shown in bold had *p* < 0.2 in univariable analysis and were therefore considered for multivariable modeling. Continuous variables are presented as mean ± standard deviation if normally distributed, and median with interquartile range (IQR) if not normally distributed. Categorical variables are presented as count/total (percent). * BEecf: base excess on the extracellular fluid.

**Table 3:** Multivariable logistic regression analysis of predictors of non-survival in dogs and cats with traumatic injuries at hospital admission.

Variables	Regression Coefficient	Standard Error	Odds Ratio	95% CI	*p*-Value
Age (month)	0.016	0.005	1.016	1.007–1.025	<0.001
ATT score	0.368	0.114	1.445	1.156–1.806	0.001
Blood pH	−0.609	0.214	0.544	0.358–0.828	0.003
Ionized calcium (mmol/L)	−0.603	0.216	0.547	0.358–0.836	0.003
Non-surgical treatment	1.086	0.244	2.963	1.836–4.780	<0.001

Note: Blood pH and ionized calcium were rescaled to 0.1-unit increments for interpretation of the odds ratios.

**Table 4:** Comparison of physiological and clinicopathological parameters in animals with surgical indications: Surgical vs. Non-surgical treatment groups.

Parameter	Surgical Treatment (*n* = 83)	Non-Surgical Treatment (*n* = 40)	*p*-Value
ATT score	6 (4–8)	6 (5–8)	0.127
**Blood pH**	**7.28 (7.20–7.35)**	**7.235 (7.14–7.31)**	**0.042**
**Lactate (mmol/L)**	**2.9 (2.10–4.60)**	**4.95 (2.80–8.82)**	**0.001**
**Bicarbonate (HCO_3_^−^) (mmol/L)**	**17.6 (15.00–20.10)**	**15.35 (11.10–20.15)**	**0.041**
**Blood Urea Nitrogen (mg/dL)**	**30 (23.75–41.25)**	**43.5 (33.25–54.00)**	**0.004**
**Albumin (g/dL)**	**2.9 (2.60–3.30)**	**2.6 (2.30–2.80)**	**<0.001**
**Type of surgical indication**			**0.034**
**Multisystem**	**30/83 (36.14)**	**18/40 (45.00)**	
**Abdomen**	**7/83 (8.43)**	**5/40 (12.50)**	
**Integument**	**5/83 (6.02)**	**1/40 (2.50)**	
**Maxillofacial**	**3/83 (3.61)**	**5/40 (12.50)**	
**Orthopedic**	**17/83 (20.48)**	**7/40 (17.50)**	
**Thoracic**	**10/83 (12.05)**	**4/40 (10.00)**	
**Urogenital**	**11/83 (13.25)**	**0/40 (0.00)**	
**Outcome**			**<0.001**
**Survival**	**67/83 (80.7)**	**6/40 (15.0)**	
**Non-survival**	**16/83 (19.3)**	**34/40 (85.0)**	

Note: Parameters with *p* < 0.05 are indicated in bold. The *p*-value for surgical indication type was derived from the chi-square test comparing the distribution across surgical indication categories. Continuous variables are presented as mean ± standard deviation when normally distributed and as median with interquartile range (IQR) when not normally distributed. Categorical variables are presented as count/total (percent).

## Data Availability

The data supporting reported results were collected from Kasetsart University Veterinary Teaching Hospital (KUVTH), Thailand, and are not publicly available due to institutional privacy policies.

## Supplementary Materials

The following supporting information can be downloaded at https://www.mdpi.com/article/10.3390/vetsci13050474/s1, Table S1. Comparison of available baseline characteristics between included and excluded trauma cases; Table S2. Bootstrap assessment of coefficient stability for the multivariable logistic regression model (1000 resamples).

## Abbreviations

The following abbreviations are used in this manuscript:

Abbreviations	Definition
ALT	Alanine aminotransferase
ASA	American Society of Anesthesiologists
ATT	Animal Trauma Triage score
AUC	Area Under the Curve
BCS	Body Condition Scores
BEecf	Base excess of the extracellular fluid
BUN	Blood Urea Nitrogen
CI	Confidence interval
CSU-CAP	Colorado State University Canine Acute Pain Scale
CSU-FAPS	Colorado State University Feline Acute Pain Scale
KUVTH	Kasetsart University Veterinary Teaching Hospital
MGCS	Modified Glasgow Coma Scale
OR	Odds Ratio
pH	Potential of Hydrogen
ROC	Receiver Operating Characteristic curve
