# Supplementary material for: Animal Trauma Triage (ATT) Score and Clinical Determinants of Survival in Dogs and Cats with Traumatic Injuries in Thailand
Source: Vet Sci. 2026 May 14;13(5):474. doi: 10.3390/vetsci13050474 (PMC13211664; doi:10.3390/vetsci13050474)
Supplement: Supplementary file 1 [file vetsci-13-00474-s001.zip › vetsci-4240902-supplementary.pdf]

Supplementary Table S1. Comparison of available baseline characteristics between included and excluded trauma cases.

Variable	Included n=184	Excluded n=183	p-value
Body weight (kg)	4.4(3.1-9.6)	4.2(3-9.4)	0.488
Age (months)	36(14-84)	28(10-65)	0.07
Time to present (min)	230(107.75-662.75)	288(123-728)	0.492
ATT score	6(4-8)	3(2-6)	<0.001
Hospitalization (min)	1366(385.75-5946.25)	277(150-497)	<0.001
Cost (bath)	15857(5402-34838.75)	5624(2890-14162.5)	<0.001
BCS	5(4-6)	5(4-5)	0.234
Species			0.717
Dogs	84/184(45.65)	87/183(47.54)	
Cats	100/184(54.35)	96/183(52.46)	
Sex			0.322
Female	82/184(44.57)	91/183(49.73)	
Male	102/184(55.43)	92/183(50.27)	
Prior treatment			0.002
No	103/184(55.98)	130/183(71.38)	
yes	81/184(44.02)	53/183(28.96)	
**Pain score**	2(2-3)	2(1-2)	<0.001
**Type of trauma**			0.226
Blunt	140/184(76.09)	129/183(70.49)	
Penetrate	44/184(23.91)	54/183(29.51)	
**Condition of trauma**			<0.001
Single trauma	62/184(33.70)	106/183(57.92)	
Multiple trauma	122/184(66.30)	77/183(42.08)	
**Treatment**			0.366
Surgical treatment	83/184(45.11)	74/183(40.44)	
Non-surgical treatment	101/184(54.89)	109/183(59.56)	
**Surgical indication**			0.557
Yes	123/184(66.85)	117/183(63.93)	
No	61/184(33.15)	66/183(36.07)	
Preexisting disease			0.594
Yes	38/184(20.65)	42/183(22.95)	
No	146/184(79.35)	141/183(77.05)	
Outcome			<0.001
Survival	119/184(64.67)	129/154(83.77)	
Non-survival	65/184(35.33)	25/154(16.23)	

Supplementary Table S2. Bootstrap assessment of coefficient stability for the multivariable logistic regression model (1,000 resamples).

Variable	Original Coefficient	Bootstrap Mean	Bootstrap SD
Age (per month)	0.016	0.018	0.006
ATT score	0.368	0.418	0.149
Blood pH (per 0.1 unit)	-0.609	-0.693	0.268
Potassium	-0.316	-0.361	0.305
Ionized calcium (per 0.1 mmol/L)	-0.603	-0.699	0.257
Hematocrit	-0.051	-0.059	0.031
Blood Urea Nitrogen (BUN)	0.004	0.005	0.010
Albumin	0.426	0.525	0.640
Prior treatment	0.283	0.352	0.563
Pain score 2	-0.452	-0.497	0.880
Pain score 3	0.187	0.331	1.022
Pain score 4	-0.204	-0.203	1.033
Type of trauma: penetrate	0.450	0.497	0.606
Non-surgical management	2.172	2.479	0.668

Note: Blood pH and ionized calcium were rescaled to 0.1-unit increments for interpretation of the odds ratios.
